# Collectanea Medica, Consisting of Anecdotes, Facts, Extracts, Illustrations, Queries, Suggestions, &c.

**Published:** 1815-04

**Authors:** 


### COLLECTANEA MEDICA,

CONSISTING OF

### ANECDOTES, FACTS, EXTRACTS, ILLUSTRATIONS, QUERIES, SUGGESTIONS, &c.

RELATING TO THE

*History or the Art of Medicine, and the Auxiliary Sciences.*

---

***Exposé of the State of Medicine in France during the year* 1814, *being the Anniversary Discourse of* Baron Des Genettes, *as President of the Faculty of Medicine of Paris.***

GENTLEMEN,

A YEAR has elapsed since one of our number, according to custom, opened the schools of medicine, by taking a view of the progress of medical knowledge during the preceding year.

The circumstances of the world were then such as have rarely occurred in the annals of nations. All the military forces of Europe, humbled so many years by our triumphs, began to drive us back upon our own frontiers, innumerable armies subsequently passed them, and after many sanguinary battles, they arrived at the gates of this capital, into which, however, they did not penetrate, until peace was restored to France, and to nearly the whole world.

Under such embarrassing circumstances, Gentlemen, you never shut up your schools nor interrupted your functions. Uniting, almost all of you, the practice of the art to the teaching of its theories, you in a manner multiplied yourselves to assist the victims of war, and to lavish your cares on our countrymen, and upon strangers, who are no longer enemies.

Those who have preceded me in this chair, have introduced the most interesting subjects: they have exhausted all that can be said as to the extensiveness of our plan of instruction.

I shall therefore confine myself to the exposition of our labours; afterwards do homage to the memory of such of our colleagues as have been recently taken from us; and lastly, fix the public attention of their fellow pupils on such of our students as shall receive the prizes this day awarded.

Baron Boyer, our colleague, has published in the course of the year a "Treatise on Chirurgical Diseases and Operations." The author of this important work thinks that the progress of modern surgery has been so rapid and remarkable, that it seems to have attained the highest degree of perfection of which it is susceptible. The greater part of surgical diseases are now perfectly well known, both with respect to their phenomena or the indications which they present. We may even frequently ascertain their proximate causes, determine their essential character, and consequently the best best treatment. Instruments and apparatus are simplified from day to day; and the application of medicaments is better understood.

Therapeutics and Hygiena, which sometimes of itself supplies their place, and always aids them, belong to both branches of the medical art; the improvement of the therapeutic and hygienic methods, therefore, equally ensure the success of internal and external applications; a new proof, among many others, of the indispensible necessity of rendering common to medicine and surgery the same institutions, and consequently the same sources of instruction.

Improvements so great, admitted by the whole world, and the chief glory of which is cheerfully conceded to the French, have rendered necessary the new publication of an entire body of chirurgical doctrine, to unite, in a treatise of a size at once convenient and elementary, knowledge scattered in books which would form a library.

M. Boyer, after the example of some of the restorers and promoters of the art, divides surgical pathology into two great parts. The first is dedicated to the diseases which appear in every part of the body, and is subdivided into several chapters, in which he treats of inflammation in general, of abscess, gangrene, burns, wounds, tumours, ulcers, and the various diseases of the bones and articulations.

The second part, drawn up after an arrangement purely anatomical, embraces the diseases peculiar to each organ. Most of the diseases which require surgical operations are referred to this division.

The author informs us, that it never was his intention to annex a complete treatise on operations, which would require immense labour. For these he refers to particular works on operative surgery, more especially to that recently published by M. Roux, adding, that the union of this work with his own will form a complete system of surgery.

On witnessing this association of a man of consummate experience with one who is still young, let those who are ignorant, or pretend to be so, of the state of our present method of instruction, cease to indulge in absurd declamations! Let them no longer repeat, in the face of direct evidence, that our school has never formed, and never will form, surgeons. We shall answer them by telling them that Richerand, Depuytren, and Desormeaux, were seated on the benches of this school, before they sat among its professors; and that the former students of this school, such as Ribes, Tartra, Marjolin, Baffos, Beauchêne, Murat, Beclar, Baren, Brechet, and several others, promise to be the worthy successors of those who are now at the head of French surgery.

On reading M. Boyer's work, as well for my own instruction, as to give an account of it at this meeting, I could not refrain from making some reflections on the chapter entitled "On the Gangrene produced by Freezing." An eye-witness of the disasters which attended the retreat from Moscow, I had an opportunity of observing, in an almost innumerable mass of men, the effects of the most rigorous cold.

I pass over in silence that perfidious sleep which fixes the body on a frozen bed, and induces inevitable death.

The doctrines of M. Boyer are entirely conformable to my own observations. To describe our sufferings would be to copy his whole chapter. Let us merely quote a few lines: "A sudden augmentation of very intense cold," says M. Boyer, "particularly when it is accompanied with wind, frequently occasions gangrenous affections and sudden death." We saw all this but too well verified, when, on the shores of the Beresina, a very violent north wind covered our faces with flakes of snow. The thermometer then stood at 21° below the freezing point; and it fell a few days afterwards to 24°, and even to 27°.

In another place M. Boyer says: "It has been thought that the cold extinguished the vital action, merely by coagulating the animal fluids; but the phenomena which accompany freezing announce that the cold acts on the solids also, and particularly on the vessels and nerves. It acts on the former by diminishing and even extinguishing their organic action; on the latter by blunting their sensibility, and thus preventing the exercise of their functions." We may go farther, and indicate a mode of action of cold, little known, a very intense effect on the brain and nerves, even when congelation has not attacked any part of the body at a distance from the centre of circulation. We have seen men, marching with every appearance of muscular energy, and the most decided and soldier-like pace, and heard them suddenly complain that a thick veil was covering their eyes. These organs, at first for an instant haggard, soon became immoveable; all the muscular apparatus of the neck, and more particularly the *sternocleido-mastoidei* muscles, became rigid, and gradually fixed the head on the right or left shoulder. The rigidity next extended to the trunk; the lower extremities then tottered, and the unhappy victim fell upon the ground, exhibiting, to complete the frightful picture, all the symptoms of catalepsy or epilepsy.

But to return to M. Boyer and his work: he has not entered into any details respecting the history of the art; he has not even indulged in a quotation, for this would not accord with his plan. "He is anxious (I copy his own expressions) to show what is proper to be done, and not what has been done, upon this or that occasion."

A professor, who, like M. Boyer, has been long in the practice of lecturing, must occasionally find his own ideas in circulation. He has, in fact, recognised some, and has informed the public of them, that he might not pass for the plagiarist of those who have actually copied from him.

In a word, the work of which we have just given a slight sketch, is worthy of M. Boyer's high reputation.

Professor Richerand has also published, in the course of the year, the sixth edition of his Physiology. Although not essentially tially different from the former editions, and, although the same arrangement is preserved, yet several points of doctrine have undergone modifications, corrections, and important additions. Some articles, which were formerly too concise, have received the desired extension. Taking advantage, in short, of the labours which daily enrich physiology, M. Richerand has learned how to found them, and to appropriate them to himself, so as to justify the title of "New Elements," which his celebrated work bears, the preceding editions of which have been already translated into several languages.

M. Alphonsus Leroy, another of our colleagues, has published a little treatise "On the Contagion which lately raged among the Cows, Oxen, and even the Human Race, in some Parts of France." He has therein discussed the causes of contagion, with the means of remedying and preventing them; and concludes by some reflections on the utility of extensive public lazarettos.

M. Leroy confesses that his work was hastily drawn up, on the spur of the moment, arising from the alarm of an approaching contagion which threatened the department of the Aisne, where, fortunately, however, it did not break out. The treatise is as it were a detached portion of an extensive work which presents ideas in many respects new, but which are not yet arrived at the degree of maturity to which the author proposes to extend them.

M. Leroy, in his ardour for the advancement of medical science, and more particularly that branch which relates to the health of the female sex, has had the boldness to advise, in a case of schirrous uterus, the revival of the operation first practised by Osiander, and since executed by M. Depuytren with all the dexterity for which he is distinguished. I merely indicate these subjects, leaving it to our colleagues to give the interesting details to the public, if they think proper.

Our colleague, M. Petit Radel, continues to be occupied in editing several articles for the *Encyclopedie Methodique.* We know that almost the whole of the surgical part of this grand dictionary has been executed of late by this laborious professor. We shall find, in succeeding numbers, several medical articles, by M. Petit Radel; among others, those which relate to hereditary, moral, nervous, organic, rheumatic, soporific, and syphilitic diseases. We shall also therein find the article "Ancient and Modern Physicians, as well religious as atheistical."

It could not be difficult for our colleague to prove the religious character of several of our professional brethren, but has he shewn with equal clearness that others among them have merited the reproach of atheism? Is not this rather one of those outrages inconsiderately committed upon great minds by the intolerance of ignorant sectaries, or by the interested supporters of the most ridiculous superstition? We see, on the contrary, that, even in the days of polytheism, the religion of the physicians, in conformity to reason, rose above vulgar credulity. How exemplary and beautiful was the worship of Galen, when, exploring the remains of men and animals, the structure of which he was studying, he said to the gods: "I shall describe these wonders, and they shall serve as the hymns by which I shall celebrate your power!"

The alarm, more or less well founded, relative to the introduction into the capital of a contagious fever, which raged in several places where the armies were quartered and fought, or left sick behind them, gave rise to instructions which were drawn up by the Faculty of Medicine. I shall respect the modesty of those who drew up these papers, and omit, like themselves, to publish their names.

Many physicians and students of our faculty have been sent to give assistance to the menaced and actually diseased departments. Others have been of great service in the hospitals of Paris, while several have fallen victims to their humane exertions. Brilliant rewards have signalized the names of part of the survivors, and all have acquired just claims on the public gratitude.

Among the inaugural dissertations presented to the Faculty in the course of the year, the following have been particularly remarked. I cite them in the chronological order in which they appeared.

1. Analytical Researches on various Affections, in which the Skin presents a Blue Colour, and in particular on those which have been designated by the name of Cyanose. By M. Gintrac.

2. Dissertation on the Plague, or Adeno-Nervous Fever. By M. Le Breton, of Rhodosto in Greece.

3. Observations intended to resolve the Question: Is the Apoplexy which is produced by an Afflux of Blood to the Brain, susceptible of Cure? By M. Riobé.

We shall now speak of the labours of the Members of the Society of Medicine, which we purposely confound with those of the Faculty of Medicine itself.

M. Alibert has given a new edition of his Elements of Therapeutics and Materia Medica. This work, which was translated as soon as it appeared into several languages, has been long diffused and received among the most celebrated medical schools of Europe. Nothing can be more judicious than the course pursued by the author in this imperfect work, which is the result of much profound and well-directed study.

The Preçis of M. Alibert respecting Mineral Waters has this year received many important additions.

His great work on the Diseases of the Skin sustains its reputation. The ninth number, which treats of syphilitic affections, is full of striking matter. The rare perfection which we see in the execution of the plates, gives a great interest to those objects, hideous as they are. We expect with a lively impatience the continuation of this magnificent work, in which scrofulous cutaneous diseases will be treated.

M. Roux has published a "Memoir and Observations on the immediate Union of the Wound after the circular Amputation of the Limbs in their continuity, and particularly after the Amputation of the the Thigh," which were read to the class of Physics and Mathematics of the Institute on the 21st of March, 1814.

This paper, the object of which is to discuss and revive, from observations actually made, methods which have been rejected, opposed, or laid aside, has been sufficiently appreciated, and has received the encouragement which it deserves, in a report by Messrs. Percy and Deschamps, adopted by the Institute, and published at the end of their Memoirs and Observations for the Year.

M. Roux has added a curious case of a Divergent Strabismus of the right Eye, cured in an adult, who was affected with it from infancy.

We are of opinion that concerning a deformity in an organ so sensible as the eye, not only are repeated experiments requisite, but there must also be a permanency in the relief or the cure, ere we can decide upon the merit of the method, and ascertain, if we may be allowed the expression, what is truly specific.

Since the publication of the work just announced, M. Roux has made a visit to London, and we are assured that he intends to publish some observations in order to make us acquainted with the particular practice of the most eminent surgeons of that capital. Such communications must be very instructive when coming from enlightened and impartial men, who have had time to look calmly around them. It will be at once advantageous and pleasing to know, after so long an interruption of our intercourse, who are now-a-days the successors or rivals of Pott, John Hunter, Keate, and Blizard.

M. Nysten has published "A Dictionary of Medicine, and of the accessory Sciences," 1 vol. 8vo. This work cannot fail to be very useful to students.

And why should we omit an important work composed by a young Spanish physician, who graduated with our Faculty, and, by the circumstances of the times, became in some measure one of our adopted sons?

M. Orfila, the gentleman in question, has this year published a "Treatise on Poisons from Minerals, Vegetables, and Animals." This general toxology is drawn up with a due regard to physiology, pathology, and medical jurisprudence. Such a work, put forth with the assistance of several experiments and accurate observations, ought to be the better received, as it was really a desideratum in medicine and jurisprudence: in fact, all preceding treatises are, without exception, incomplete, inaccurate, and far behind the present state of our knowledge.

M. Orfila, in the first place, carefully describes the physical and sensible characters of poisons, in their simple and natural state; and afterwards indicates the chemical properties of those substances, noting with great exactness the phenomena which they exhibit by means of the greatest possible number of re-agents.

M. Orfila afterwards shews the differences which poison, when mixed with food of various kinds, presents with the same re-agents.

But

But what most concerns our art and the public security, is the indication of the best methods of arresting the action or incipient effects of poisons, and of remedying the disorders which they have already produced on the animal economy.

We think it right to observe, that we have promoted, of late years, to the degree of Doctor of Medicine, several Spaniards of the greatest promise. They have sustained in the midst of us, by the lustre of their talents, a valuable portion of the national honour; while the majority of their fellow countrymen, full of the recollection of the valorous achievements of their ancestors, have broken the yoke which an execrable tyrant sought to impose on them.

The Bulletin of the Faculty and of the Society of Medicine, the editing of which is confided to Professor Dumeril, has communicated to the world, more or less in detail, whatever has been most interesting to the profession. We may remark in particular, in a recent number, the extract of an observation on the ligature of the external iliac artery, communicated by M. Bouchet, chief surgeon to the Hotel Dieu of Lyons. This fact, with the manner in which it was brought forward, and the reflections which it occasioned in the Society, remind us of the most flourishing days of the old Royal Academy of Surgery.

I must now call your attention to the losses which we have experienced. We have already observed, that the faculty of medicine, and the society formed in its bosom, are indissolubly knit together. It is thus, as a member of the society which forms part of our school, that I shall express our common regret for the loss of one who honoured me with his particular regard, Charles Louis Dumas. When, upon the death of Grimaud, cut off from his professional career at the age of thirty-seven, it was you, Dumas, who placed yourself in the ranks to succeed him; you saw, without regret, the crown adjudged to Fouquet, who, at the age of sixty-five, could no longer have any rivals. Another chair became vacant by the death of M. Sabatier, and the keeper of the seals of France, who honoured you with his esteem, wished you to fill it. Afraid, lest it should appear that you were indebted to favour, and not to your own merits, you scrupled not to re-descend into the arena.

The writings of Dumas may be classed under three different heads. The first belong to the institutions or elements, and are purely anatomical or anatomico-physiological; the second relate to practical medicine; and the third class is composed of various isolated pieces, such as memoirs, observations, discourses, and eloges.

We shall take a cursory view of these various productions, not according to the division which we have established, but in chronological order, which will best exhibit the progress and history of his mind.

Dumas made his debut in 1785, at the age of twenty only, by an an inaugural dissertation upon "Life." This first performance indicates the direction which his mind took and constantly preserved from that moment.

A short time afterwards, he was a candidate for a prize proposed by the Royal Society of Medicine. It was required "to determine in what species, and at what period, of chronic diseases, fever may be useful or dangerous, and with what precaution we ought to excite or moderate it in their treatment." The memoir published by Dumas on this occasion is a judicious selection of the opinions of the best authors. Before the age of experience, we can only resolve similar questions in this way. The prize was divided between Dumas and M. Pujol, formerly a practitioner at Castres in Languedoc.

Dumas published, for the competition of the year 1790, to which we have alluded, a Series of Theses, which, conformably to the custom of the faculty of Montpelier, were composed, distributed, and sustained, within the space of fifteen days.*

In

* Quæstiones medicæ duodecim, propositæ pro regiâ cathedrâ vacante per obitum D. Joannis Sabatier, quas propugnabit triduo integro mane et seró, diebus 4, 5, et 6, mensis Novembris anni 1790, in augustissimo Ludovicæo Monspeliensi Carolus-Ludovicus Dumas.—Quæstio I. Quantum contulerit schola Monspeliensis in promovendis chimiæ túm philosophicæ, túm practicæ, progressibus?—Quæst. II. An quodlibet morbi genus à causâ sibi propriâ excitetur, ita ut tot diathesium, quot morborum genera diversa admitti debeant?—Quæst. III. Quare navigatores morbis exanthematicis, febribus, etc., in Europâ laborantes, iis sub æquatore et in Americâ, absque ullo artis auxilio, subitò quasi liberentur, reduces verò in Europam ipsis rursùs corripiantur, et vice versâ?—Quæst. IV. Quid ex vasorum lymphaticorum cum textu cellulari connexione, ex absorbente illorum officio et structurâ, censendum sit de humorum in ipsis vasis motu retrogrado, et per textum cellularem transitu?—Quæst. V. An doctrina veterum circa crises et dies criticos sit constans et certa? Quonam fundamento innitatur dissensus auctorum circa hanc doctrinam?—Quæst. VI. An detur legitima suppurationis distinctio? Quid ad therapeiam conferat? Num ex illâ canones practici deduci possint ad felicem ulcerum curationem?—Quæst. VII. An dentur morbi, et quinam á veteribus medicis descripti nunc evanidi?—Quinam verò nostrâ ætate vigentes veteribus ignoti?—Quæst. VIII. De magnetismo animali, de somnambulatione et somnambulorum vaticiniis judicium medicum.—Quæst. IX. An in morbis acutis satius sit crises congruis auxiliis antevertere, quâm ægros crisium periculo exponere?—Quæst. X. Febrium in genere theoriam et therapeiam exponere.—Quæst. XI. An omninò respuenda sit, et è foro medico dejicienda consideratio influxûs planetarum et siderum; nempè phasium lunæ, dierum canicularium, solsticiorum et equinoxiorum recursûs in corpus tùm sanum, tùm ægrotum? An potiùs in dubiis comprobetur observationibus

In 1791, Dumas gave an edition of the "Complete Course of Fevers," of the late De Grimaud, in 4 vols. 8vo. conformably to an autograph copy specified in the testament of the estimable author. Dumas was anticipated a few months or weeks by a self-elected editor, who, without delicacy, published the same work in three volumes only, and in the same form. The opinions of medical men are divided as to the merit of the two editions.

Dumas was associated the same year with M. Petit D'Arsen, in translating "An Essay on the Nature and Treatment of Pulmonary Consumption;" written in English by Thomas Reid. This work contains only one fact and its inductions. This fact is, that the vomiting arising from sea-sickness has been known to give relief in incipient phthysis. The inductions are, that repeated emetics, in small doses, might be useful in the treatment in the same disease, and in the same stage. Without discussing the merits of the original work, we may affirm, that the translators, by adding a preface and notes, have succeeded in making a far better book.

Elected professor of Anatomy and Physiology in 1795, Dumas published in 1797, in 1 vol. 4to. a Treatise on Myology, after the manner of Winslow, Albinus, and Chaussier. This work is entitled, "Methodical System of the Nomenclature and Classification of the Muscles of the Human Body, with Descriptive Tables for recalling their Ancient Names, their New Names, Situation, Attachments, Direction, Composition, Figure, Connection, and Use." A dictionary containing the whole *synonymy* of the muscles terminates this treatise. It is an imitation and an extension of the work of one of our colleagues, whom we have mentioned constantly, preceded by general principles and reflections on the formation of languages.

Dumas published, in 1800, and in 4 vols. 8vo. his Principles of Physiology, or Introduction to the Experimental, Philosophical, and Medical Knowledge of the Living Man." He brings forward with address whatever was most important on this subject, grounding his opinions in preference upon the writings of Stahl, Haller, and Grimaud. Another person, who approached in some respects the first, who, in point of erudition, equalled the second, and who surpassed the third in every respect, (I mean Barthey,) was offended at the silence of Dumas, who, in borrowing several of his ideas, had forgotten to acknowledge their author. Barthey had a right to complain, and he did so, with that air of authority which was peculiar to him, and which he but too strictly maintained in this discussion.

I pass over slightly the "Physiological Sketch on the Transformation of the Organs."—*Journal de Physique,* 1805 and 1806.

---

observationibus quod non exigui sit momenti talis consideratio? —Quæst. xii. Utrum et quâ ratione partium tùm solidarum, tùm molliùm regenerationem ars promovere, juvare, coercere, præpedire queat?

To

To believe that the stomach can fulfil the functions of the brain, and, *vice versâ,* the brain those of the stomach, would be to return to the chimerical ideas of Paracelsus and Van Helmont. It was quite in a different spirit that Dumas published an observation, that does great honour to his talents, on the subject of an epilepsy rendered intermittent, and afterwards cured by the administration of bark.—*Journal de Medicine.*

Dumas pronounced, in 1804, a Discourse on the future Progress of the Science of Man. His hopes were founded on the continuation of the application of analysis, and the perfection of the faculty of observation.

A new edition of his Physiology, as before in 4 vols. 8vo. appeared in 1806.

The Eloges of Fouquet and Dorthey, published the first in 1807, and the second in 1808, are two papers which do equal honour to the head and heart of Dumas. He had, it is true, in praising the former, merely to repeat what medical Europe had proclaimed for thirty years ; but he had to make known in the latter a man nearly of the same age with himself, a former competitor for the same chair, and a rival for glory in the same school; although Dorthey, in a more especial manner, cultivated other branches of the natural sciences, in which he has left a great name.

We now come to a period of his life when Dumas published a work more original, and more peculiarly his own, if I may be allowed the expression, than any of the foregoing. It is the best of his productions, and also the last.

The treatise of Dumas on "Chronic Diseases," is divided into four parts. In the first, he explains the essential phenomena of these diseases, and the differences which exist between them and acute diseases. The second part explains the theory of the formation of chronic diseases. In the third part, he examines the modifications produced in diseases, by age, sex, passions, and influence of climate. The last and fourth part exhibits the application of the distinction of the elementary affections to the treatment of diseases.

This work, although very extensive, remained imperfect until it was followed by a volume of developments and applications to a great number of observations. When Dumas wrote this treatise, which appeared at Paris in 1812, he enjoyed a high reputation, which could only be increased by it: he was surrounded by that consideration which his talents and his eminent social qualities merited. Finally, he was loaded with academical, literary, and other honours of every kind, when he died at Montpelier, on the 3d of April, 1813, at the age of 47.

We have still another heavy loss to deplore, and it is still more recent and more premature; we mean that of Julien-Jean-Cæsar Le Gallois, who died in February last.

Professor Dumeril has already published a very interesting account of Le Gallois The son of an honest agriculturist in easy circumstances, he received a liberal education: he gave early signs

 of of talent, and, on finishing his studies, he felt a *penchant* for medicine, which he studied in the university of Caen, which reckoned, among its medical professors, Chibourg, Le Canu, and Rousell, and which had produced Vicq-d'Azyr, Thouret, and Vauquelin. The revolution about this period assumed a most frightful shape. Those who still retained some sentiments of commiseration and some ideas of equity, and the young in particular, burning with indignation, ranged themselves under the standard of a party which has since been distinguished—that of *Federalism*. Le Gallois became, under these circumstances, one of the leaders of the students. It is useless, impolitic, and perhaps dangerous, to dwell upon those times of calamity: suffice it to say, that the party in question was crushed in Calvados as well as throughout France, and that Le Gallois, obliged to fly, hid himself first at Paris, where he was discovered; that he took refuge among the sciences, and was so fortunate as to be employed in the manufacture of saltpetre in a department at a distance from the capital. Upon the formation of the three schools of medicine he returned to Paris, where he was received as one of the pupils from the departments; among his fellow students, he was distinguished, and began by fixing the attention of the learned world upon him by his Thesis for the doctorate on the following question: "Is the blood identically the same in all the vessels which it passes through?" This production announced a man of science, who was determined to proceed in his studies by the thorny, but otherwise fertile, road of experience.

Le Gallois shortly afterwards took part in the discussions occasioned by the famous thesis of Boulet, who, in an ingenious and erudite paradox, threw some doubt on the existence of Hippocrates.*

Suddenly,

---

* This thesis was entitled, "Dubitationes de Hippocratis vita, patria, geneologia, forsan mythologicis; et de quibusdam ejus libris, multo antiquioribus vulgo quam creditur, Dissertatio medico-historica, quam sistit et tueri conabitur die secunda mensis pluv. an. XII. præside Petro Sue, J. B. J. Boulet [Hesdinensis] Medicus Insulanus, &c." The examiners were Messrs. Thouret, Lereux, Petit Radel, Des Genettes, and Dumeril. The candidate was remarkable by a most decent and modest exterior, and an easy and brilliant elocution. He was, besides, advantageously known as one of the chief editors of Dessault's Journal de Chirurgie.

Le Gallois published soon afterwards, in the Journal General de Medicine, an article with the title of "Chronological Researches concerning Hippocrates." Admitting that M. Boulet had directed his attack with great skill and erudition, Le Gallois concluded that Hippocrates lived during the Peloponesian war; first, because no writer, previous to that war, had made any mention of him; secondly, because some points of his doctrine are manifestly

Suddenly, a grand idea struck Le Gallois and absorbed all the faculties of his mind. He sought for the solution of the boldest problem,—for he sought nothing less than the discovery of the principle of life!

The history of the sciences exhibits to us the first chemists as almost all occupied for centuries with the transmutation of the metals and the universal panacea. They could neither create gold nor prolong the life of men, and yet they enriched the arts with numerous useful processes, and medicine with several very powerful remedies.

Le Gallois did not succeed any more than they in determining in what life precisely consists, and, perhaps, it is not given to the feeble intelligence of man to discover the primordial laws of the great phenomena of our organization; but, in seeking for the solution of a question still undecided, Le Gallois threw great light on several very important points in physiology. He is, in this respect, the most distinguished man which our school has produced since Bichat.

The labours of Le Gallois are contained in a work entitled "Experiments on the Principle of Life, particularly on that of the Motion of the Heart, and on the Seat of this Principle." This valuable collection of facts has produced a work equally important; viz. the report made on this subject to the first class of the Institute, by Baron Humboldt, so dear to the sciences on many accounts, and our colleagues, Hallé and Percy; these gentlemen caused to be repeated before them, first, the series of experiments relative to the principle of the inspiratory movements; secondly, the experiments relative to the principle of the powers of the heart. But these subjects cannot be analyzed in the present discourse.

Le Gallois has also left a work on the teeth of the rabbit and the guinea-pig, on the duration of gestation in the latter animal, and on the relaxation of the symphysis pubis at the moment of parturition. The observations and experiments on these various subjects were made while Le Gallois was enquiring into the principle of life.

The result of all his enquiries and experiments relative to circulation are also printed in the excellent article Heart, (*Cœur,*) which Le Gallois supplied for the New Dictionary of the Medical Sciences, which reckons among its authors several professors of this

---

manifestly borrowed from authors who lived during, or shortly before, that war; thirdly, because authors, whose testimony is irrefragable, and who wrote as well during as after the war in question, say expressly that he lived at that era, quoting on this head circumstances which signify the same thing; fourthly, because the dialect which he used refers to the same æra; fifthly, because the objection deduced from astronomical observations which are given as a peremptory proof of his great antiquity, is a mere futility which would not deserve to be seriously refuted, if several persons had not ascribed to it a false importance.

faculty, faculty, and almost all the most distinguished physicians and surgeons of this capital.

Le Gallois, who was qualified by his education and talents to practise either surgery or medicine, adhered to the latter branch of the healing art. He had been nearly a twelvemonth physician to the Bicetre. He lived in Paris, and it was when proceeding on foot to his duty, as he frequently did, that he was attacked by a peripneumonia, to which he fell a victim in the beginning of February last year, leaving an interesting family inconsolable for his loss.

We have to express our regret, that we cannot detain you a few minutes longer by detailing at full length the life of Villars, dean of the faculty of medicine of Strasburg, and associate of our society of medicine, who died on the 27th of June last.

The details of his life would exhibit to your view a man deprived almost from birth of all hope of acquiring the slightest knowledge of letters. You would see afterwards by what difficult bye-paths, and how, with talents almost entirely flowing from nature, he attained an honourable place among the physicians, and particularly the most distinguished botanists, of the day.

The intimacy of Villars, in his youth, with J. J. Rousseau; his zeal for the instruction of the numerous pupils whose minds he formed; his humane attentions to the sick as physician to a great military hospital, or a practitioner among all classes of society, will present some affecting traits to whoever shall draw up his elogé with all the extent it deserves.

Let us no longer withhold the recompenses which await those, who at the last exhibition of our practical school ought to be preserved as examples for the emulation of their fellow students.

This last exhibition has not been in truth so numerously attended as on other occasions, and the candidates have not given the same proofs of proficiency as in preceding years. He whose name was first called, was a convalescent from a severe disease contracted in the hospital, when he underwent his examination. The other candidates who will receive prizes after him, whatever be the place which they occupy are equally worthy of encouragement and indulgence, both on account of their exertions and of the arduous circumstances in which they found themselves at the end of the last and the beginning of the present year.

Let us all now forget the violent and too long continued misery from which we have but just emerged, and listen to the consolations of peace, which can alone soften down our past misfortunes.

But what have we not to expect, gentlemen, from the advancement of medical knowledge, under the reign of a prince who finds so many beautiful examples in every page of the history of the kings his ancestors and predecessors. The establishments which Louis IX. Francis I. Henry IV. Louis XIV. Louis XV. and Louis XVI. consecrated to letters and to humanity, are ever present in the memory and engraved on the heart of Louis XVIII. He has come among us to restore and to preserve. Surrounded by the councils of wisdom, he will preserve for us whatever is good, and will improve whatever can be made better.

NOTE,

NOTE.—We have given this translation somewhat shortened of the French exposé. Our wish was to show the customs of their Institutes, which, we conceive, may be followed with advantage. At the same time, it is impossible not to perceive the meagreness of information deriveable from apparently so many sources. Is it because the French wish to arrogate every thing to themselves that all the information they could derive from English medicine is the translation of a book now almost forgotten, and never held in much estimation among us (Dr. Thomas Reid on Consumptions)? —In our last we noticed the greater clearness of the practical remarks given by our English medical writers above the French. We shall now present the instance to which we referred, and add a physiological fact in confirmation of our other remark.

"Most writers who have employed the term Black Cataract, attach the same signification to it as to Amaurosis; and Sauvages gives them as synonymous terms.

"Morgagni, to whom we refer in all questions of pathological anatomy, never met with an example of black cataract, and considers amaurosis and this to be the same disease. 'Longum de amaurosi sermonem habui sive de cataracta nigra. Nunc paulo breviorem de suffusione.' *De Sed. et Causis*, Epist. xiii. No. 14.

"The German writers confound the two diseases. If we examine the treatises of Maitre Jean, St. Yves, Gendron, and others, on diseases of the eyes, we find that cataracts may be of various colours—white, yellow, blue, green, brown, and black; but, as if they had forgotten the fact, speak of nothing but the white.

"Doubts of the existence of black cataract have been expressed even by surgeons of the first eminence, who refer every thing that has been said respecting it to the amaurosis. Mr. Tartra, a candidate for the chair vacated by the death of Mr. Sabatier, says, that we have not a single authentic example of the disease;* but in a recent instance, at the Hospital la Charité, the crystalline after death was found perfectly black, and was seen by upwards of one hundred persons. An old woman, blind upwards of twenty years, died of serous apoplexy. The last fifteen years of her life, she could not distinguish the greatest light from total darkness. On examination of the eyes, the right was withered, and the cornea opaque through its whole extent. The left eye was in its natural state, the cornea transparent, the pupil round, large, and of its natural appearance. The eye was opened, and the crystalline was perfectly black. It was firm and solid; its superficial laminæ were transparent. When dried, it was divided into two portions: one, the central, black like ink, hard, and solid, constituting three-fourths of its bulk; the other formed a thin transparent envelope to the preceding, of a chesnut colour. The capsule was in its natural state. A diseased state of the membranes, as in amaurosis,

"* Concours pour la chaire de Med. Operat de l'Operation de la Cataracte. Thes. de M. Tartra, p. 28. Paris."

existed, existed, which sufficiently accounts for the impossibility of distinguishing light from darkness.

"This is not a solitary instance. Many persons who were supposed to be affected with amaurosis, and were condemned to perpetual blindness, recovered their sight by the skill of operators who recognized the real disease.* Mr. Wenzel's Treatise contains the following case.

"'My father having been sent for to Vienna, in the year 1760, to give advice to the Empress-Queen, who had a considerable relaxation of the eye-lid (of which she was soon cured), operated, during his stay in that city, on the General-Marichal Molck, the pupils of whose eyes had no motion, and the crystallines were so black, that both the celebrated Van Swieten and De Haen imagined his disorder to have been a gutta serena. As, however, it appeared to my father, after the necessary questions, and a due examination of the eye, that the operation was likely to succeed, the General determined to submit to it. The cornea, and the anterior capsule of the eye, first operated upon, were scarcely divided, when the crystalline escaped through the incision with great velocity, fell at some distance from the patient, and broke into two parts. Upon examination, it was found to be almost black, firm, and of the consistence of plaster. The crystalline of the second eye came out entire; my father taking care gradually to drop the upper lid, in proportion as the incision of the cornea advanced, in order to prevent its sudden explosion. This was as black as the first, much more solid, and almost stony. The General had no bad symptoms after the operation, and in the usual course of time recovered his sight.'

---

"* M. Pelier relates a case of this kind. 'An old man, named Jean Brunet, was blind for the space of fifteen years. The eyes were examined with attention, and the smallest alteration in appearance could not be discovered. The pupils were handsome and black, and the iris contracted as in health. His sight had gradually failed, without any pain, but occasionally he saw floating objects before his eyes. As the disease proceeded, the flame of candles at a distance seemed like as many shining revolving suns. From this history, I concluded the blindness depended on opacity of the crystalline, and my conjectures were increased, as he could distinguish light from darkness. I extracted the lens from each eye, and the sight was immediately restored. The crystallines, when extracted, were found very thick, and black as ink, which induced all the preceding oculists to say his disease was amaurosis, and consequently incurable.'—*Pelier, Recueil de Memoires & Obs. sur les Maladies de l'Œil*, page 227. 1783.

"It is important to remark here the circumstances on which Pelier founded his opinion:—1st. The patient perceived objects floating before his eyes, as flies, cobwebs, &c. a phenomenon almost constant in the commencement of cataract, but rare in amaurosis. 2d. He distinguishes night from day."

 "Under

"Under the article Cataracte, of the Encyclopedie Methodique, and of the Dictionaire Opthalmologique, both written by the same author, we find the following paragraph:

"'Cataracte noire. Etat particulier du chrystallin, qui en perdant sa transparence, acquiert une *couleur brune* tirant beaucoup sur le noir; cette espece de cataracte n'est pas aussi aisée a decouvrir que celle que l'on traite pour l'ordinaire, cependant la *couleur de la chrystalline est differente de la couleur noire* que l'ouverture de la pupille offre naturellement.'

"In the two cases related by this author, the iris had lost its contractile power.

"If the recorded cases of black cataract are rare, it is, probably, because it has been confounded with amaurosis. No difficulty can exist in distinguishing these two diseases, where it is complete, as the latter is then accompanied with *total blindness and inability to distinguish night from day*, which is different in cataract.

"The state of the iris affords no criterion, as, in the case of Wenzel's cataract, it was motionless, while instances of amaurosis exist with perfect contractility of that muscle. The suddenness of the formation of amaurosis can be no criterion, for cataract has been produced in a few hours.* But it is deserving of remark, that patients with cataract see better in a dim than in a strong light, from the corresponding enlargement of the pupil, while the reverse obtains in amaurosis."—*Journale de Medicine.*

The diagnostic marks contained in the last paragraph relate equally to other cataracts as to the black, and are noticed by Mr. Stevenson among the characters of the disease in its various forms. But the accuracy and minuteness of our English oculist in other more important distinctions, will be acknowledged by every impartial reader.

"There is one species, fortunately very rare, denominated by Baron de Wenzel *black* cataract, accurately to distinguish which from some other disorders of the eye, requires a more than ordinary share of experience. Mr. Pott refers to it, when he says, 'sometimes the whole crystalline is dissolved into a fluid, still preserving its transparency; which kind of alteration forms what is by some called one species of gutta serena, by others *black* cataract.'† Nothing less than the high character of Mr. Pott, and his extensive opportunities of observation, would have satisfied me that he had actually met with such a solution of the lens. It is certainly, however, not constant in the *black* cataract; in which case, the crystalline generally possesses a considerable degree of firmness. Baron de Wenzel goes so far as to assert, that in a case of this sort he extracted 'the lens, which was found almost black, firm, and of the consistence of plaster; that before the operation, the pupils had *no motion*, and the crystallines appeared so

"* Tenon, Memoire sur la Cataracte.

"† Pott's Remarks on Cataract, note, p. 7."

black, that both the celebrated Van Swieten* and De Haen imagined his disorder to be gutta serena.'† He says, 'that it is not easy to distinguish a black cataract from a gutta serena;' but that 'though the difference in the appearance of the eye in those two disorders be small, it may be distinguished by a careful observer, since the diseased crystalline has always a peculiar appearance, unlike to that of the bottom of the eye.'‡ Mr. Ware, in a note to the Translation of the Baron's Treatise, p. 52, observes, 'though it cannot be denied that a cataract sometimes exists in an eye whose colour is dark, yet this darkness is very different from the clear black appearance which the pupil has, not only when the eye is in a state of health, but also when it is affected with a true simple gutta serena.'

"As this is unquestionably a point of the greatest importance, I shall subjoin a few other particulars, by which I think its character may be better understood.

"The condition of the eye, which seems to have given rise to the appellation *black* cataract, is as follows. There are changes in the crystalline by which the transmission of light is so little obstructed, that the pigmentum may be discerned through it; yet, the rays emanating from external objects, and striking upon the cornea, are so irregularly refracted in their passage through the altered structure of the lens, as to represent a very indistinct picture upon the retina. The disease is characterized by a dark bluish or slate colour in the centre of the lens, somewhat resembling wrought unpolished iron, the circumference retaining more transparency. In these instances, there is still a degree of *oblique* vision, except when the patient is exposed to a strong glare of light; in which state, from the contraction of the iris, the rays can only pass through the thickest portion of the crystalline. This description of cataract can only be recognised by the above symptoms, by the slightly turbid appearance in the centre of the eye, and the generally inactive state of the iris, but most of all, by the images of external objects not being reflected, as in the sound organ, whilst to the inexperienced the structure may seem perfect, from the pupil still retaining a dark complexion. This kind of cataract is too often confounded with impaired sensibility of the optic nerve, or retina, constituting gutta serena; and the miserable sufferer is consigned to darkness for life, under an erroneous idea that the operation could be of no avail. But this is so far from the truth, that I do not hesitate to recommend the operation in all cases of the above description, where the features of the person

"* See Morgagni de Causis et Sedibus Morborum, epist. xiii. p. 207, vol. i. where we find the term *black* cataract applied, as it commonly was by the Germans, to a palsy of the optic nerve.

"† Translation of Baron de Wenzel's Treatise on the Cataract, case iii. p. 51 and 52.

"‡ Ibid, case vi. p. 58."

who who examines the eye are not reflected. Let the following case serve as an illustration.

"A lady, upwards of fifty, applied for my assistance, on account of a great defect in her sight. She had, for ten years, consulted oculists of considerable and deserved celebrity, who, fully impressed with the belief that the symptoms depended upon some paralysis of the optic nerve, contented themselves with prescribing such medicines as they conceived were best adapted to increase its sensibility. On a careful examination of the eyes, I found reason to suspect that the case was an instance of what has been called black cataract, and encouraged the hope of restoration to sight by an operation. As this was the first time she had heard her complaint called cataract, she complied with my proposal as a forlorn hope. She was, however, most agreeably disappointed, the operation proving completely effectual; and she is now capable of reading, by the help of proper glasses, the smallest print with the greatest ease and comfort.

"The success of the above case has given me courage to pursue the same plan in a few other instances of a similar nature, and with the same happy result."—*Stevenson on Cataract*, 2d edit. p. 37.

Want of room obliges us to defer our remarks on some cruel experiments made in Paris on living animals, which might have been spared by an attention to our English physiologists.

